# Assessing skin cancer histopathology reporting against minimum dataset standards in a low-resource setting

**DOI:** 10.1186/s12913-025-13965-2

**Published:** 2025-12-27

**Authors:** Sari Taha, Samia Hamad

**Affiliations:** 1 An-Najah Global Health Institute (GHI), An-Najah National University, P.O. Box 7, Nablus, Palestine; 2 Division of Public Health, Faculty of Medicine and Allied Medical Sciences, An-Najah National University, P.O. Box 7, Nablus, Palestine; 3 Department of Medicine, Faculty of Medicine and Allied Medical Sciences, An- Najah National University, P.O. Box 7, Nablus, Palestine

**Keywords:** Audit, Melanoma, Basal cell carcinoma, Squamous cell carcinoma, Skin cancer, Quality improvement

## Abstract

**Background:**

Structured pathology datasets enhance communication and management by improving clarity, completeness, and accuracy. This study aimed to audit pathology reports of skin cancer in Palestine.

**Methods:**

Comprehensive, time-driven sampling was employed by reviewing all pathology reports of melanoma, basal cell carcinoma (BCC), and squamous cell carcinoma (SCC) from January 2021 to December 2024. The data included sociodemographic variables and the core items published in the dataset for the histological reporting of skin cancers by the Royal College of Pathologists. A completion rate of 90% was selected as the standard of measurement.

**Results:**

None of the included 113 reports documented all items. The macroscopic items were completely reported in 25% and 39.1% of BCC and SCC reports, respectively. For both BCC and SCC, specimen type was reported for all cases, and clinical site was missing in one case of each. The lesion margins were recorded in most reports of BCC (68.7%) and SCC (67.4%). However, macroscopic lesion dimensions were absent in 51.6% of BCC reports and 48.4% of SCC reports. The other items were not mentioned in any report. Only clinical site, specimen type, and the three dimensions of the specimen were included in all melanoma reports. The histopathological subtype was mentioned in only one report.

**Conclusions:**

Skin cancer reports did not document most core items. Implementation of standardized reporting is limited by resource constraints and suboptimal health information system. However, transition from narrative to structured reporting requires the development of national regulations and organizational policies, supported by committed leadership. Exploratory research is recommended to identify the barriers to implementation of structured reporting.

## Introduction

Skin cancers are broadly classified into two major types: melanoma and non-melanoma skin cancers, which are mainly categorized into basal cell carcinoma (BCC) and squamous cell carcinoma (SCC) [1]. Findings from the Global Burden of Disease study (GBD) indicate a rise in the prevalence of skin cancers between 1990 and 2017, with increases of 161% for melanoma, 310% for SCC, and 77% for BCC [2]. The burden of skin cancer varies considerably across populations with a higher prevalence among those with fair skin tones exposed to intense UV radiation [3]. In 2022, for instance, the age-standardized prevalence was estimated at 37.9 per 100,000 population in Australia, compared to only 0.37 per 100,000 population in China [1]. Among populations with darker skin tones, skin cancer is often diagnosed at later stages, which is associated with a worse prognosis [4, 5].

Accurate pathology reporting of skin cancer is a key element of case diagnosis, management, and prognosis [6]. Therefore, standardizing cancer reporting by using pathology datasets are increasingly adopted by pathology departments worldwide [7]. A pathology dataset is a structured template prepared for consistent, accurate, and clear histopathological reporting [7, 8]. These templates facilitate synoptic reporting by defining data elements, standardizing nomenclature, and outlining measurement methods [8]. Synoptic reporting was initially defined as a two-column format, with the first column containing a predefined data element (e.g., ‘maximum diameter of the specimen) and the corresponding pathologist-reported value (e.g., 4 mm) in the second [8, 9]. The systematic development of pathology reporting was described by Srigley et al. through an incremental, six-level staging framework. At level one, pathology reports are unstructured, relying on free-text narratives, whereas at level six, reports are structured based on a standardized, electronically implemented dataset [10]. Globally, most pathology departments conform to level three, at which standardized checklists are used but responses to these checklists remain unstandardized and reliant on free-text inputs [7, 10].

The use of pathology datasets enhances clarity, completeness, and accuracy [7, 11, 12]. At the patient level, the checklist exercise is, in itself, a reminder to capture essential histopathological information whose omission could compromise patient care [13]. Along the care continuum, these datasets facilitate communication between pathologists and clinicians [14–16]. Importantly, diagnostic accuracy of pathology reports can be improved if pathologists are provided with clinical images of excised lesions, especially facilitated by the increasing use of telemedicine [17]. At the system level, structured, standardized reporting facilitates the use of data for monitoring, evaluation, policymaking, quality improvement, research, and artificial intelligence applications [14–16].

Several national and international protocols have been developed for guiding skin cancer reporting [6, 18–20]. In 2019, the Royal College of Pathologists updated and published datasets for histopathological reporting of melanoma, BCC, and SCC. The datasets recommend a list of core and noncore items to be included in pathology reports to enhance diagnostic and prognostic accuracy. The core items describe essential items, such as clinical history, nature of the specimen, and other macroscopic and microscopic elements [20–22].

Implementation of standardized, pre-structured reporting in Palestine faces several challenges. Histopathology remains a developing field, with recent developments in practice, education, and training. In 2021, only four public histopathology laboratories and six histopathologists served a population of over three million, amounting to a rate of only two pathologists per million people [23]. The limited availability of human resources, combined with time constraints, may lead to prioritization of diagnostic accuracy over comprehensive reporting. This is in addition to the lack of essential pathological tools such as immunohistochemical and molecular testing required for accurate diagnosis of skin cancer.

Pathology datasets provide a standard reference against which clinical audits can evaluate cancer reporting. A clinical audit is a systematic examination of a clinical process or outcome against predefined, evidence-based standards, aiming to inform the implementation of change to improve the quality of care [24, 25]. Given the importance of histopathological reporting for effective diagnosis, prognosis, and management of skin cancers, audits can identify reporting problems in Palestine, guiding quality improvement projects against local challenges. This study aimed to assess histopathology reporting of melanoma, BCC, and SCC in Palestine.

## Methods

### Study design and settings

This retrospective study evaluated pathology reporting of melanoma, BCC, and SCC in a major Palestinian hospital. Data were collected between November 2024 and January 2025 from reports written between January 2021 and December 2024. The hospital name was anonymized per regulations. The study followed the Plan, Do, Study, Act (PDSA) cycle framework [26, 27], and employed a process-based approach, assessing the reporting process rather than the clinical outcomes due to the delayed and time-consuming measurement of the latter. This approach is based on the hypothesis that the process of improving reporting and streamlining communication can enhance skin cancer diagnosis and management, as outcomes. The cycle plan addressed the change concept, purpose statement, target healthcare workers, location, and timeline. A completion rate of at least 90% was set as the standard of measurement and aim of change [28].

### Sampling and data collection

This study used comprehensive, time-driven sampling, including all melanoma, BCC, and SCC pathology reports from January 2021 to December 2024. The Royal College of Pathologists recommends a minimum of 20 cases for a whole-department audit [28]. The reports were retrieved using diagnostic codes for each type of cancer. Mucosal cases of melanoma, BCC, and SCC, including vermilion lip cancer, were excluded [22, 29]. The retrieved data included sociodemographic variables, such as age and sex, and the core items included in the dataset for the histological reporting of skin cancers by the Royal College of Pathologists [20–22]. For melanoma, core items included clinical items (site of origin, type of biopsy, lymph nodes status); macroscopic items (dimensions of specimen, maximum diameter and elevation of lesion, atypical features of asymmetry, borders, color…); and microscopic items (histopathological subtype, thickness, ulceration, mitotic index, lymphovascular invasion, satellites, neurotropism, growth phase, tumor-infiltrating lymphocytes, regression, margins, staging, and coding). For BCC and SCC, some items, such as ulceration and regression, were not included as core items [20–22].

### Data analysis

Data were inserted into an Excel spreadsheet (Microsoft Excel, 2016), separately for each type of cancer and imported into IBM SPSS (version 24.0). Age was summarized using mean and standard deviation. The frequencies and proportions were reported for the categorical variables, including the demographic information, clinical sites, the filling rate of each core item, and the rate of completed reports. A report was considered complete if all core items were adequately described according to the adopted dataset. The Mann-Whitney test was used to assess for an association between the number of reported core items and the year of the report. Statistical significance was set at a *p*-value < 0.05.

### Ethical considerations

The Institutional Review Board (IRB) of An-Najah National University (ANNU) reviewed and approved the study (Ref: Med Sept. 2024/11). The Ministry of Health granted approval for accessing pathological reports. The data were stored safely on a computer protected by a secured password and could be accessed only by the researchers. These data were solely used for research purposes.

## Results

### Basal cell carcinoma

A total of 64 BCC reports were included and 1 report was excluded. The mean age of patients was 63.9 years (*SD* = 12.2). None of the reports documented all core items (Table 1). The macroscopic items were completely reported in 25% of the reports, whereas no report fully included the clinical and histological items. Specimen type was reported for all cases, and clinical site was reported for all but one case. All reports documented either two or three dimensions of the specimen; however, only 37 documented all three dimensions required as a core item. Macroscopic reporting of maximum lesion dimension was absent in over half (51.6%) and microscopic reporting of margins was absent in nearly one-third of the reports (31.3%). Moreover, no reports included the maximum clinical dimension, histological subtype, thickness, level of invasion, maximum histological dimension, or pTNM staging. Of the four reports of basosquamous cell carcinoma included, only one included information about lymphovascular invasion. All reports used the implemented ICD system, in which an item is selected from a predefined list, whereas the SNOMED coding was not adopted. The median number of reported items was 4 (IQR = 3–4), with no significant association with the year of the report (*p* = .886).

**Table 1:** Reported core items in basal cell carcinoma reports

Core item	Reported	Unreported
**Clinical data** *n* (%)		
Clinical site	63 (98.4)	1 (1.6)
Maximum clinical dimension/diameter	0 (0.0)	64 (100.0)
Specimen type	64 (100.0)	0 (0.0)
**Macroscopic description** *n* (%)		
Dimension of specimen	37 (57.8)	27 (42.2)
Maximum dimension/diameter of lesion	31 (48.4)	33 (51.6)
**Histological data** *n* (%)		
Histological subtype	0 (0.0)	64 (100.0)
Thickness	0 (0.0)	64 (100.0)
Level of invasion	0 (0.0)	64 (100.0)
Perineural invasion	1 (1.6)	63 (98.4)
Margins	44 (68.8)	20 (31.3)
Maximum dimension/diameter of lesion	0 (0.0)	64 (100.0)
pTNM	0 (0.0)	64 (100.0)
SNOMED code	0 (0.0)	64 (100.0)

### Squamous cell carcinoma

A total of 46 SCC reports were included and 2 reports were excluded. The mean age of patients was 66.0 years (*SD* 24.5). None of the reports included all core items (Table 2). The macroscopic items were reported in over one-third of reports (39.1%), whereas no report completely included the core clinical or histological items. Specimen type was reported for all cases, whereas clinical site was absent in only one report. All reports documented either macroscopic two or three dimensions of the specimen, with only 32 recording the three dimensions required as a core item. Most reports recorded the maximum macroscopic dimension (51.6%) and the lesion margins (67.4%). However, no report described the maximum clinical dimension, histological subtype, thickness, level of invasion, maximum histological dimension, and pTNM staging. The median number of reported items was 5 (IQR = 4–6), which was not significantly associated with the year of the report (*p* = .293).

**Table 2:** Reported core items in squamous cell carcinoma reports

Core item	Reported	Unreported
**Clinical data** n(%)		
Clinical site	45 (97.8)	1 (2.2)
Maximum clinical dimension/diameter	0 (0.0)	46 (100.0)
Specimen type	46 (100.0)	0 (0.0)
**Macroscopic description** *n* (%)		
Dimension of specimen	32 (69.6)	14 (30.4)
Maximum dimension/diameter of lesion	26 (56.5)	20 (43.5)
**Histological data** *n* (%)		
Histological subtype	1 (2.2)	45 (97.8)
Grade	43 (93.5)	3 (6.5)
Thickness	0 (0.0)	46 (100.0)
Level of invasion	0 (0.0)	46 (100.0)
Perineural invasion	0 (0.0)	46 (100.0)
Lymphovascular invasion	0 (0.0)	46 (100.0)
Margins	31 (67.4)	15 (32.6)
Maximum dimension/diameter of lesion	0 (0.0)	46 (100.0)
pTNM	0 (0.0)	46 (100.0)
SNOMED code	0 (0.0)	46 (100.0)

### Melanoma

Only three cases of melanoma were reviewed, with a mean age of 80.3 years (SD = 7.2). The clinical items, including the clinical site and specimen type, as well as the three dimensions of the specimen, were mentioned in all reports. One report did not record the maximum dimension of the lesion, and another report did not record the lesion margins. The histopathological subtype was mentioned in only one report. However, none of the other core items were recorded in any report.

## Discussion

Standardizing and structuring pathology reports are essential for improving the diagnosis and management of skin cancer, and enhancing communication between clinicians [6]. This retrospective study reviewed pathology reports of melanoma, BCC, and SCC to assess reporting adequacy against the minimum dataset developed by the Royal College of Pathologists. The findings revealed that most core items were inadequately reported. Macroscopic items, such as specimen dimensions and maximum lesion diameter, were documented more frequently than clinical and microscopic core items. Microscopic dimensions and lesion margins were sometimes reported, albeit less frequently.

This study demonstrated low compliance with reporting the core items outlined in the selected minimum dataset. Two published national audits of skin cancer reports were conducted in the UK using the minimum dataset published by the Royal College of Pathologists in 2002, an earlier version of the dataset than that used in the present study. While these audits were conducted in high-income settings where standardized reporting is encouraged and often implemented, both audits reported inadequate compliance, with fewer than one-third of reports documenting all core items [30, 31]. These findings align with other pathology audits conducted across income settings and cancer types [32–38]. Even when structured reporting was recommended in Nigeria, in settings more comparable to those of the present study than in the UK, the adoption of structured reporting remained minimal and the change was statistically nonsignificant [32]. These findings highlight the challenges of implementing standardized, pre-structured reporting, particularly in resource-limited settings, where financial, technical, motivational, and legal barriers may limit policy development, implementation, and compliance [39, 40].

In this study, clinical site, specimen type, and specimen dimensions were the most frequently reported items. Few other items, such as microscopic dimensions and margins of lesion, were less reported, whereas most core items were entirely omitted. These include items that are essential to manage BCC and SCC, such as the subtype and depth of invasion. This variability between items is consistent with other audits evaluating cancer reporting, where microscopic items, such as level of invasion and staging, were frequently missing, while margins were more often documented [30, 35, 37]. This pattern may reflect a perceived hierarchy of importance among pathologists, with specific items considered more critical for diagnosis and management than others, and that some items, such as staging, can be deferred to clinicians.

In addition to the scarcity of human and physical resources in Palestine, implementing an advanced reporting framework may be technologically infeasible given the current state of the health information system (HIS). While HIS is adopted in most healthcare facilities, its functionality is largely limited to electronic patient records [41]. Further expansion and optimization of HIS face multiple challenges, including limited funding, poor interoperability, inadequate training and organizational support, and low staff digital competency [42–44].

These context-specific limitations add to other cultural and institutional barriers to implementation of standardized, pre-structured reporting that are commonly described by pathologists across settings, including resistance to change, lack of education and training, the need for financial incentives, and perceptions of increased workload [39, 40, 45, 46]. Resistance to change may be attributed to a professional culture rooted in established narrative‑reporting habits and a preference for free-text formats over structured templates, which are often viewed as inflexible [39, 40]. Others may perceive this change as adding to their workload, as reported in studies conducted in Belgium and the Netherlands [39, 40]. This is despite the organizational and economic efficiency of standardized, pre-structured reporting, including cost-effectiveness, improved report creation and interpretation, and data retrieval for research and quality control [7, 11, 12, 14]. In the Palestinian context, the lack of human, financial and physical resources, in addition to health system governance limitations and overriding healthcare needs, may amplify these universal challenges. However, given the severe lack of local pathology research, qualitative, exploratory studies are needed to identify the cultural and institutional barriers to implementation and to inform context‑specific strategies for this implementation.

This audit found that pathology reporting of skin cancer is limited to level 1, relying on conventional text-based reports. Cancer Care Ontario is an instructive example of a scalable, national project aimed at improving pathology reporting in Canada [47]. While implemented in high-resource settings, certain elements of the project, such as the use of appropriate quality indicators for cyclical auditing and targeted education for underperformers, are transferable to low-resource settings. In such contexts, a transition to a synoptic-like level 3 reporting system is feasible, as its implementation is mainly driven by organizational rather than technological factors. Effective implementation requires commitment at the levels of the health system, institution, and department. This includes the development of national regulations and organizational policies, supported by committed leadership. Importantly, this transition is facilitated by the leadership of senior pathologists who recognize the value of standardized reporting in enhancing the quality of care despite concerns of time burden and workflow disruption.

The major limitation of this study is the small sample size. Only 3 melanoma and 46 SCC reports were retrieved. However, a smaller sample size allows for rapid data collection, which is key for optimizing the use of resource use, maintaining stakeholders’ engagement, and supporting effective change implementation. Unlike research studies where representativeness is prioritized for generalizability concerns, audits focus on examining actual practices and evaluating quality improvement projects. Moreover, despite the adoption of a comprehensive sampling approach, including all cases reported over four years, the small sample size suggests that a bulk of cases might have been referred to private laboratories at the request of patients or the referring clinicians. Furthermore, the dataset used in this audit includes a comprehensive list of core items, which are unlikely to be fully documented in resource-limited settings where structured reporting is not a priority on the organizational agenda. Additionally, bias may have been introduced from the lack of independent data extraction by multiple reviewers, which would have ensured inter-rater reliability. However, this audit adhered to predefined methods and standards to provide the basis for future reaudits following the implementation of changes. To the best of the authors’ knowledge, this audit is among the first national audits performed in Palestinian healthcare settings, and the first to assess pathology practices. It also contributes to the limited body of pathology research both locally and regionally.

## Conclusions

Standardization of pathology reporting of skin cancer facilitates communication between pathologists and other healthcare professionals, which is key for improving the diagnosis, management, and prognosis of skin cancer. This study audited pathology reports of melanoma, BCC, and SCC against the minimum dataset developed by the Royal College of Pathologists. The audit revealed all reports of the three types of skin cancer did not document most of the core items. Notably, the macroscopic items related to the dimensions of the specimen and lesion were more frequently documented than the clinical and microscopic items. Additionally, microscopic dimensions and lesion margins were less frequently reported. Standardization of pathology reporting at the national level is challenged by the scarcity of human and physical resources and the limitations in the health information system. Transition from free-text, narrative to a more structured reporting is feasible if the development of national regulations and organizational policies is supported by committed leadership at the department level. Further qualitative, exploratory research is recommended to identify the cultural and institutional barriers to implementation of structured reporting.

## Data Availability

The data are available from the corresponding author upon reasonable request.

## Abbreviations

BCC: Basal cell carcinoma
SCC: Squamous cell carcinoma
GBD: The Global Burden of Disease study
PDSA: The Plan, Do, Study, Act cycle
IRB: The Institutional Review Board
ANNU: An-Najah National University
